# Monooxygenase-dehydrogenase cascade for sustained enzymatic remediation of TMA in salmon protein hydrolysates

**DOI:** 10.1128/aem.01242-25

**Published:** 2026-02-03

**Authors:** Rasmus Ree, Øivind Larsen, Sushil Gaykawad, Sreerekha S. Ramanand, Antonio García-Moyano, Irina Elena Chiriac, Pål Puntervoll, Gro Elin Kjæreng Bjerga

**Affiliations:** 1 NORCE Climate & Environment, NORCE Research AS 521537, Bergen, Norway; 2 LEITAT Technological Center 202560, Terrassa, Barcelona, Spain; Kyoto University, Kyoto, Japan

**Keywords:** fish protein hydrolysate, flavin-containing monooxygenase, trimethylamine, trimethylamine-N-oxide, glucose dehydrogenase, cofactor recycling, affective testing, oxidoreductase

## Abstract

Fish protein hydrolysates hold great promise as nutraceuticals, yet their application as food ingredients or nutraceuticals is currently limited by their fish-like odor. This odor is mainly due to the presence of trimethylamine (TMA), a volatile biogenic amine resulting from the breakdown of naturally occurring trimethylamine-N-oxide (TMAO) in marine fish. The bacterial trimethylamine monooxygenase mFMO can oxidize TMA into TMAO using molecular oxygen and the cofactor nicotinamide adenine dinucleotide phosphate (NADPH). We have established an enzyme cascade that takes advantage of glucose dehydrogenase to recycle NADPH from NADP^+^, significantly decreasing the cost of the reaction and paving the way for using the enzyme system in fish protein hydrolysates targeted for human consumption. We demonstrate that the dual enzyme system works in an industrially relevant substrate. Salmon protein hydrolysate treated with an mFMO/glucose dehydrogenase cocktail showed a 75% reduction in TMA. A trained sensory panel perceived an improved odor across several parameters, including a reduction in the characteristic TMA smell.

**IMPORTANCE:**

Marine by-products are a valuable source of high-quality peptide ingredients; however, their application in the food market is limited by the unpleasant fishy odor caused by trimethylamine (TMA). An enzyme that oxidizes TMA to the odor-free trimethylamine-N-oxide (TMAO) in salmon protein hydrolysates is known, but it requires excessive amounts of NADPH, an expensive cofactor. Here, we describe a cofactor regeneration system that allows using less cofactor in the enzyme-driven TMA removal process. This dual enzyme system removed 75% of TMA from a salmon protein hydrolysate, resulting in a significantly reduced fishy odor as confirmed by a trained sensory panel compared to the untreated control. This enzyme cascade is an important step toward making targeted TMA removal economically feasible for marine biomass valorization.

## INTRODUCTION

**Fig 1:**
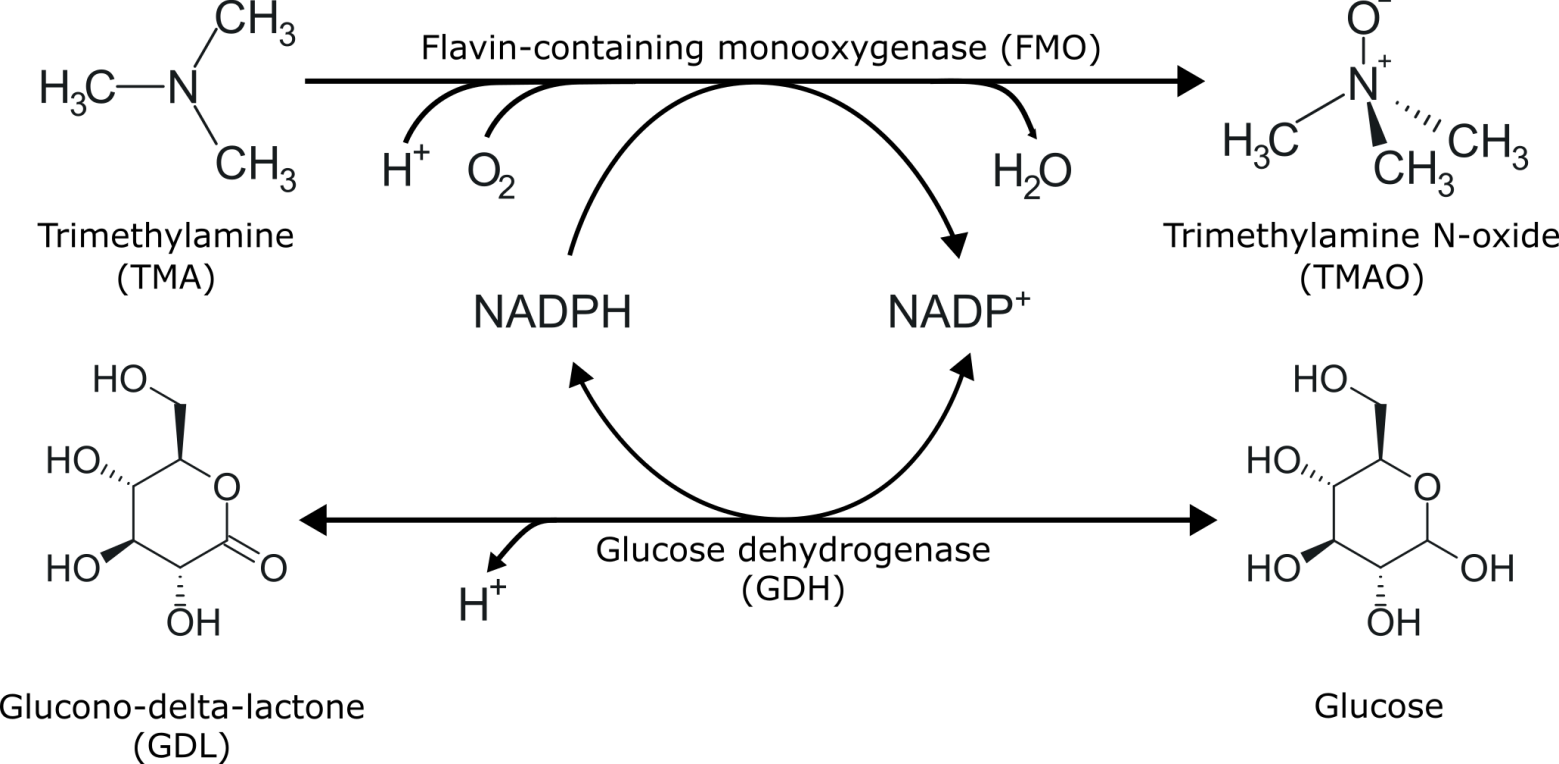
TMA oxidation and glucose dehydrogenation by FMO and glucose dehydrogenase (GDH). Scheme of the TMA-to-TMAO and glucose-to-GDL reaction cycle. NADPH and NADP^+^, reduced and oxidized nicotinamide adenine dinucleotide phosphate, respectively.

The Norwegian aquaculture and fisheries industry produced nearly 3.2 million tons of seafood in 2024 (1). Of this, close to 1.1 million tons were byproducts, defined as any product that is not the main product produced from a raw material, such as heads, frames, and backbones. Fish protein hydrolysis is a way of utilizing such byproducts through controlled proteolysis, often using subtilisins (2). The product, fish protein hydrolysates (FPH), is a mix of amino acids and small peptides that are suitable for human consumption. It is an excellent protein source, with protein content varying depending on production method; salmon byproducts typically contain 69%–89% protein (3–5). FPH has been explored as a source of bioactive peptides with antioxidant, anti-hypertensive, and anti-inflammatory effects (6, 7). However, according to statistics of Norwegian use, FPH is mainly used in pet food and feed formulations for farmed fish (1) rather than in human nutrition. The main barriers to consumer acceptance of FPH as food appear to be its fishy odors and flavors (1, 2, 8). Consequently, there is a demand for novel strategies that enable the use of these nutritious fish byproduct-derived proteins in the higher-value food market. Several compounds are known to contribute to fish odor, of which trimethylamine (TMA) is a key component. TMA is a volatile, biogenic amine that is formed postmortem from trimethylamine-N-oxide (TMAO) (8). TMAO is a naturally occurring metabolite in fish from cold and deep-sea environments and is, importantly, odorless. TMAO is believed to function as an osmolyte (9) and as a piezolyte, counteracting pressure-mediated inhibition of protein function (10, 11). After slaughtering, TMAO in fish is converted to TMA by TMAO-reducing bacteria, contributing to its fish-like odor (12–14). The FPH industry has identified TMA as a key target for improving the sensory properties of FPHs. To remove it, some industrial actors currently use nanofiltration of FPH, but this untargeted method has the drawback of causing significant protein loss and altering the nutritional composition (15). An interesting alternative to filtration is to use an enzyme to specifically target TMA. Previously, we have shown that the bacterial trimethylamine monooxygenase mFMO can be used to remove most of the TMA from salmon FPH in a targeted approach (16, 17). The mFMO enzyme is a flavin-containing monooxygenase (FMO) (18) isolated from the marine gammaproteobacterium *Methylophaga aminisulfidivorans* (19). It belongs to a family of closely related bacterial FMOs, which catalyze the oxidation of TMA (16, 20, 21). These bacterial FMOs oxidize TMA using molecular oxygen and NADPH as a cofactor, leaving TMAO and the oxidized cofactor NADP^+^ as products (Fig. 1, top) (19, 21). To enhance compatibility with industrial processing conditions, a thermostable mFMO variant, termed mFMO_20, was generated through structure-based engineering and shown to perform well at up to 65°C (17). The oxidation reaction catalyzed by mFMO uses one molecule of NADPH per molecule of TMA. As NADPH is very expensive, mFMO-assisted removal of TMA in fish protein hydrolysates will not be industrially viable unless cofactor consumption is managed in a cost-effective manner.

Two strategies have been explored to reduce the cost of cofactors in reactions that depend on them: enzyme-cofactor engineering and cofactor recycling. Enzyme engineering can be used to improve the affinity for a cheaper cofactor with the same redox function, such as nicotinamide adenine dinucleotide (NADH) (22) or nicotinamide mononucleotide (23). However, mutation may compromise other aspects of enzyme functionality, such as reaction rate and stability, requiring substantial screening to achieve a useful enzyme reaction rate (24). To our knowledge, successful attempts to change the cofactor specificity of FMOs from NADPH to NADH have not been reported. Furthermore, although NADH is cheaper than NADPH, it remains prohibitively expensive for an industrial process (25). Cofactor recycling is thus an attractive strategy for cofactor management and has been successfully implemented in several systems (25). It involves regenerating the redox cofactor, enabling its reuse in multiple reaction cycles. This can be achieved through direct chemical reductive regeneration (26), homogenous (27, 28) and heterogeneous (29) regeneration using hydrogen and organometallic catalysts, photocatalytic regeneration (30), and electrochemical regeneration (31). Alternatively, enzymatic regeneration (32–34) uses a secondary enzyme reaction, which reduces the cofactor while oxidizing a sacrificial substrate, to maintain cofactor availability and drive the main reaction. It has several advantages: it can be highly specific and, depending on the choice of sacrificial substrate, toxic components and catalysts can be avoided (25). For food production involving enzymatic TMA removal, the sacrificial substrate and products must be food safe, and the regeneration enzyme must be compatible with both the processing conditions and the TMA-oxidizing enzyme. Moreover, glucose is an inexpensive substrate, and this recycling strategy allows the use of the cheaper cofactor NADP^+^ instead of NADPH, significantly reducing costs associated with cofactor supplementation.

Various enzymes have been used to regenerate NADPH, including alcohol dehydrogenase, phosphite dehydrogenase, glucose dehydrogenase (GDH), and glucose-6-phosphate dehydrogenase (35). Phosphite dehydrogenase has been used in fusion constructs to enable sustained catalysis mediated by mFMO (36) and a Baeyer–Villiger monooxygenase (37). Like FMOs, the latter enzyme belongs to the class B flavoprotein monooxygenases (38). While GDH has not yet been reported for use with FMOs, it is an attractive cofactor-regenerating enzyme due to its widespread application, its simplicity, and the low cost of its substrate, glucose. Of note, glucose dehydrogenase B (GdhB) has been used to regenerate NADPH during enzymatic Tyrian purple production by an FMO (39). For the intended application in this study, it is important to note that both the glucose substrate and the products, glucono-1,5-lactone (also known as glucono-delta-lactone, or GDL), as well as the GDL hydrolysis product gluconate, are recognized as safe and widely accepted food ingredients (Fig. 1, bottom) (40).

In this study, we couple the activity of the thermostable mFMO_20 to the activity of the glucose dehydrogenase GdhB from *Priestia megaterium* (previously known as *Bacillus megaterium*) (41, 42). We demonstrate that this enzyme cascade, in the presence of excess glucose and the oxidized cofactor NADP^+^, effectively depletes TMA in salmon protein hydrolysate while recycling NADPH and producing GDL and its derivative gluconate. Our approach demonstrates the utility of cofactor recycling for cost reduction in TMA remediation in an industrially relevant context.

## RESULTS AND DISCUSSION

### Establishing a cofactor recycling system for a TMA monooxygenase

When setting up a cofactor recycling system for the enzymatic removal of TMA in FPH, we chose the GdhB enzyme and glucose as sacrificial substrate, and used NADP^+^ as the added cofactor. To guide enzyme dosage and cofactor concentration in the dual enzyme system, we characterized the kinetics of GdhB for glucose. The *K*_m_ of GdhB for glucose was 68.7 mM (Table S1), a factor of 8 × 10^5^ higher than that of mFMO_20 for TMA (17). The *V*_max_ was about half that of mFMO_20. To compensate for the lower efficiency of GdhB, we set up the recycling system using a glucose concentration of 50 mM and a 10:1 ratio of GdhB to mFMO_20. Since NADH is more stable and less costly than NADPH, activities of a mFMO_20 and GdhB were assessed using both NADPH and NADH as electron donors. GdhB can catalyze the oxidation of glucose by using both cofactors and can also catalyze the reverse reaction using NAD(P)H and GDL (Fig. S1A). A sufficiently high glucose concentration is thus required to drive the reaction toward GDL formation and NADP^+^ reduction. However, the activity of mFMO_20 with NADH is only 4% of its activity with NADPH (Fig. S1B), necessitating the use of NADP^+^ in our system.

**Fig 2:**
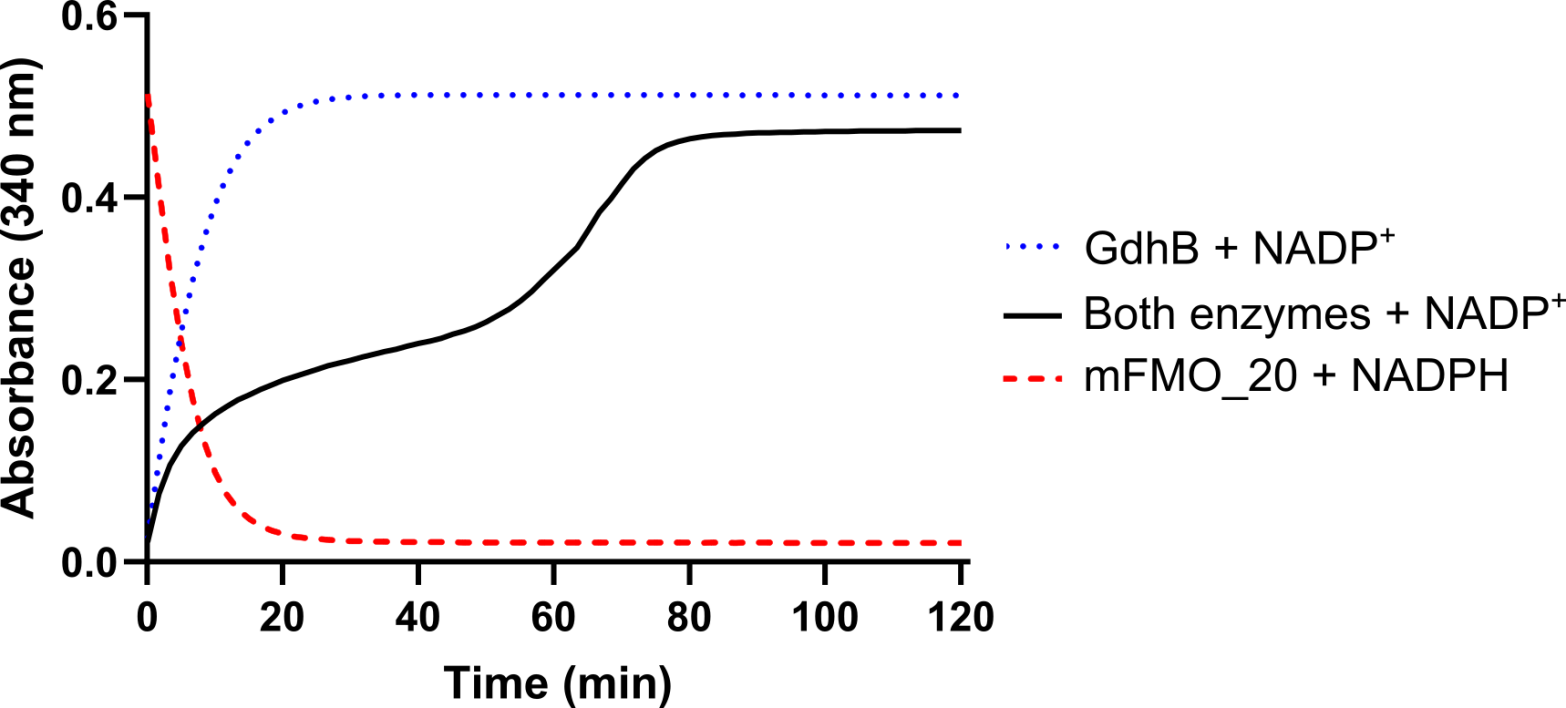
Cofactor recycling during enzyme reactions over time. Reactions were monitored by absorbance at 340 nm, indicative of NADPH concentration. Three conditions were tested: (i) GdhB alone (500 nM) with 50 mM glucose and 100 µM NADP^+^, tracking NADPH formation; (ii) mFMO_20 (50 mM) with 500 µM TMA and 100 µM NADPH, tracking NADPH consumption; and (iii) both enzymes combined with both substrates (glucose and TMA) and 100 µM NADP^+^. In the combined reaction, NADPH is regenerated through cofactor recycling between GdhB and mFMO_20. Absorbance was recorded over 120 min.

To evaluate the performance of the cofactor recycling system, we monitored a set of model reactions with mFMO_20 and GdhB individually and in combination over a 2 h time period (Fig. 2). Substrates were supplied at concentrations exceeding enzyme and cofactor levels (50 mM glucose and 500 µM TMA vs 100 µM NADP^+^ or NADPH) to minimize substrate limitation, and absorbance at 340 nm was recorded as an indicator of NADPH concentration. In reactions with either enzyme alone, NADPH was consumed or produced rapidly, stabilizing within approximately 30 min. In contrast, when both enzymes were present with their respective substrates and NADP^+^, absorbance continued to rise for more than 70 min, approaching the maximum expected for NADPH, corresponding to the starting NADP^+^ concentration (100 µM). The biphasic increase in NADPH reflects the dynamics of cofactor recycling: during the first hour, NADPH formation by GdhB is balanced by its consumption by mFMO_20, but once TMA is depleted, NADPH accumulates toward the theoretical maximum of 100 µM. Notably, the NADPH accumulation suggests that most of the 500 µM TMA was oxidized using only 100 µM cofactor, demonstrating efficient recycling between the two enzymes. These results confirm that NADP^+^ reduction and NADPH oxidation occur concurrently, and that the dual-enzyme reaction system sustains cofactor recycling for at least 80 min without apparent cofactor degradation. Enzyme and cofactor stability under extended reaction times and elevated substrate concentrations should be explored to better understand the limits of continuous operation.

**Fig 3:**
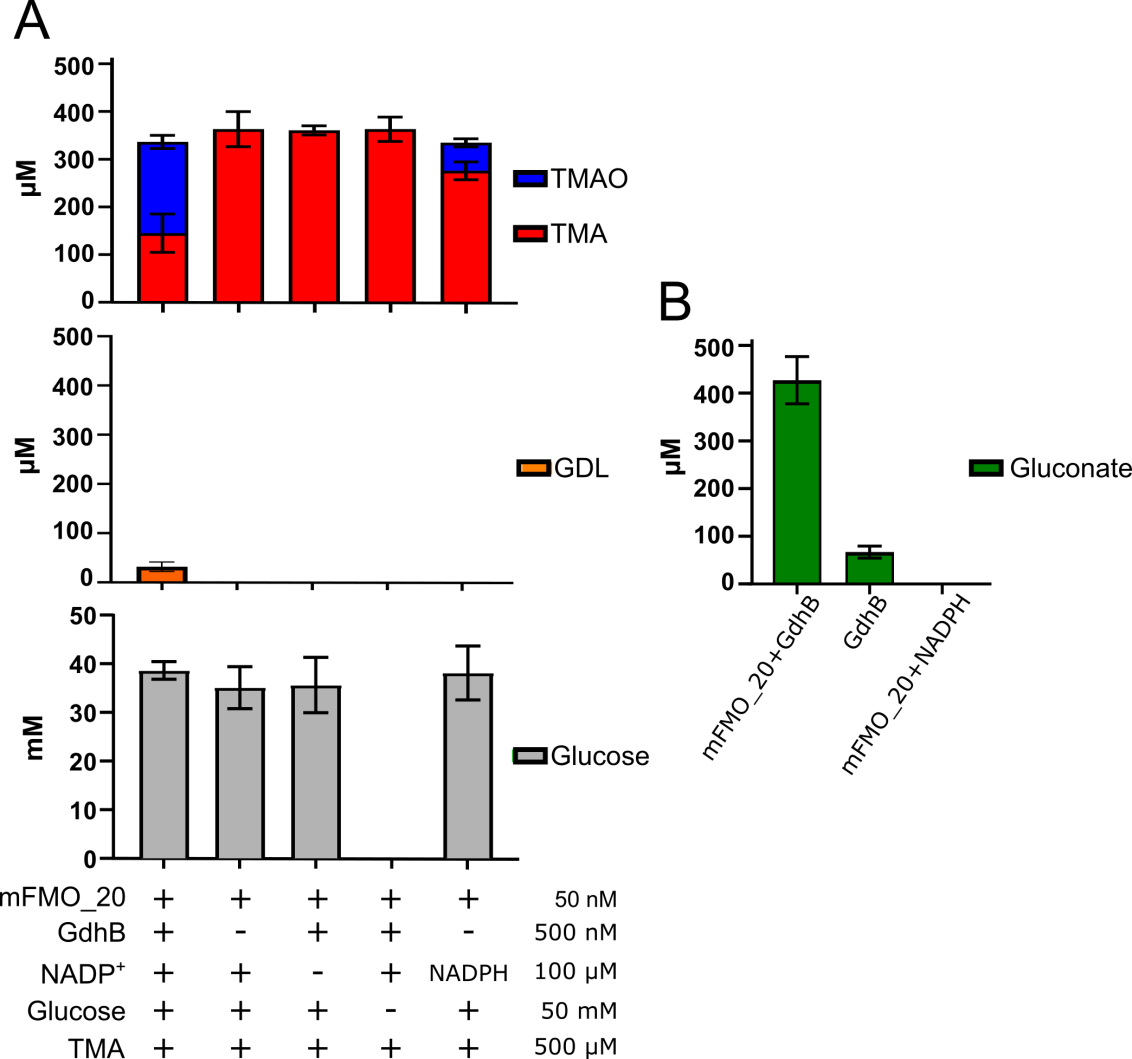
TMA oxidation driven by cofactor recycling. (**A**) Enzyme assays (*n* = 3) with the indicated components (+, present; −, absent): mFMO_20, GdhB, NADP^+^ (or NADPH), glucose and/or TMA at the given concentrations were incubated for 1 h at 25°C and analyzed by LC/MS for the presence of TMA, TMAO, glucose, or GDL, which were quantified against a standard curve. (**B**) Enzyme assays (*n* = 3) of mFMO_20 and GdhB with NADP^+^, GdhB with NADP^+^, and mFMO_20 with NADPH. All reactions contained an excess of 50 mM glucose and 500 µM TMA. Cofactor concentrations were 100 µM. Assays were incubated for 1 h at 25°C before heat inactivation of enzymes and GDL-to-gluconate conversion. Gluconate was quantified against a standard curve.

To confirm the reactions underlying cofactor recycling (Fig. 2), we quantified substrates and products after 1 h in these model reactions using liquid chromatography/mass spectrometry (LC/MS) (Fig. 3A; Tables S3 and S4). The 1-h time point was selected based on Fig. 2, where NADPH accumulation continued beyond 60 min and approached a plateau near 80 min. This interval was expected to capture an active but incomplete reaction, allowing detection of both substrates and products. As in the previous experiment, NADP^+^ was used as a cofactor to ensure that TMAO formation depended on GdhB activity, and TMA was provided in molar excess relative to the cofactor to enable assessment of recycling efficiency. The two products, TMAO and GDL, were detected when both enzymes, both substrates, and the cofactor were present. As expected, TMAO was not formed when any of the recycling components (cofactor, GdhB, or glucose) were removed. When 100 μM NADPH was used as cofactor in the absence of GdhB, only 58 µM TMAO was formed, and as expected, no GDL was produced. Cofactor recycling was further supported by the formation of 191 µM TMAO in a reaction with only 100 µM NADP^+^, consistent with the time-course data in Fig. 2. This confirms that cofactor recycling allowed oxidation of TMA in amounts exceeding the initial cofactor concentration.

Based on the reaction scheme in Fig. 1, oxidation of TMA to TMAO by mFMO_20 and dehydrogenation of glucose to GDL by GdhB are expected to proceed in parallel, with NADPH and NADP^+^ cycling between the two enzymes. Under ideal conditions, the molar amounts of TMAO and GDL should increase proportionally, reflecting the coupled redox reactions. However, LC/MS analysis revealed that GDL concentrations were substantially lower than expected (Fig. 3A). One plausible explanation is spontaneous hydrolysis of GDL to gluconate, as previously reported (40). Although this possibility was considered during LC/MS method development, no ions corresponding to gluconate were detected. To verify GDL formation under the same reaction conditions as analyzed by LC/MS (Fig. 3A), we quantified gluconate as a proxy for GDL using an enzymatic colorimetric assay. In this assay, gluconate conversion drives reduction of a proprietary probe, generating a chromogenic signal at 450 nm, which is proportional to gluconate concentration. Prior to measurements, samples were heat-treated to ensure complete hydrolysis of GDL to gluconate (43). When both enzymes were combined with their respective substrates and NADP^+^, 459 µM gluconate was detected (Fig. 3B). Removing mFMO_20 resulted in GdhB alone forming 72 µM gluconate, whereas no gluconate was detected when mFMO_20 was present with NADPH in the absence of GdhB. The 459 µM gluconate concentration is close to the starting TMA concentration, indicating that more than 90% of TMA was oxidized, which is higher than the TMAO concentration measured by LC/MS (Fig. 3A). This discrepancy likely reflects differences in workflows and analytical principles between LC/MS and the colorimetric assay. For LC/MS analysis, assay samples were mixed with acetonitrile (90%) to precipitate proteins before centrifugation. At this solvent ratio, highly polar compounds may also precipitate, as visually observed in reactions with high TMAO concentrations (data not shown). Across all conditions, measured concentrations of TMA+TMAO and glucose were approximately 15% lower than expected based on the input amounts (Fig. 3A). This suggests that precipitation during sample preparation could contribute to the underestimation of these compounds in LC/MS assays. In contrast, we do not expect similar losses in the gluconate assay, as there is no evidence that gluconate precipitates during sample preparation.

A disadvantage of using GdhB for cofactor recycling in this system is its relatively low catalytic activity compared to mFMO_20, necessitating a relatively high concentration of glucose to drive the recycling reaction. We used 50 mM glucose, which is comparable to the 100 mM glucose used in previous studies with this enzyme (41, 44, 45). In contrast, those studies used cofactor concentrations of 10–500 mM, which are 100–5,000 times higher than those used in our assays (Fig. 2 and 3). The final glucose concentration in the protein hydrolysate obtained in this study was relatively high at 1.75% wt/wt, assuming a 10% dry weight content (see next section). This level is comparable to the 1% xylose concentration used with heat to achieve browning and caramelized flavor for odor masking of salmon protein hydrolysate (46). To achieve better TMA remediation with lower levels of glucose in the final product, it could be useful to improve the activity and substrate affinity of GdhB through enzyme engineering (47) or through immobilization (48, 49). Other cofactor recycling systems that employ better-suited sacrificial substrates could be explored, or systems that utilize substrates naturally present in FPH.

### The dual enzyme system reduces TMA levels in salmon protein hydrolysates

**Fig 4:**
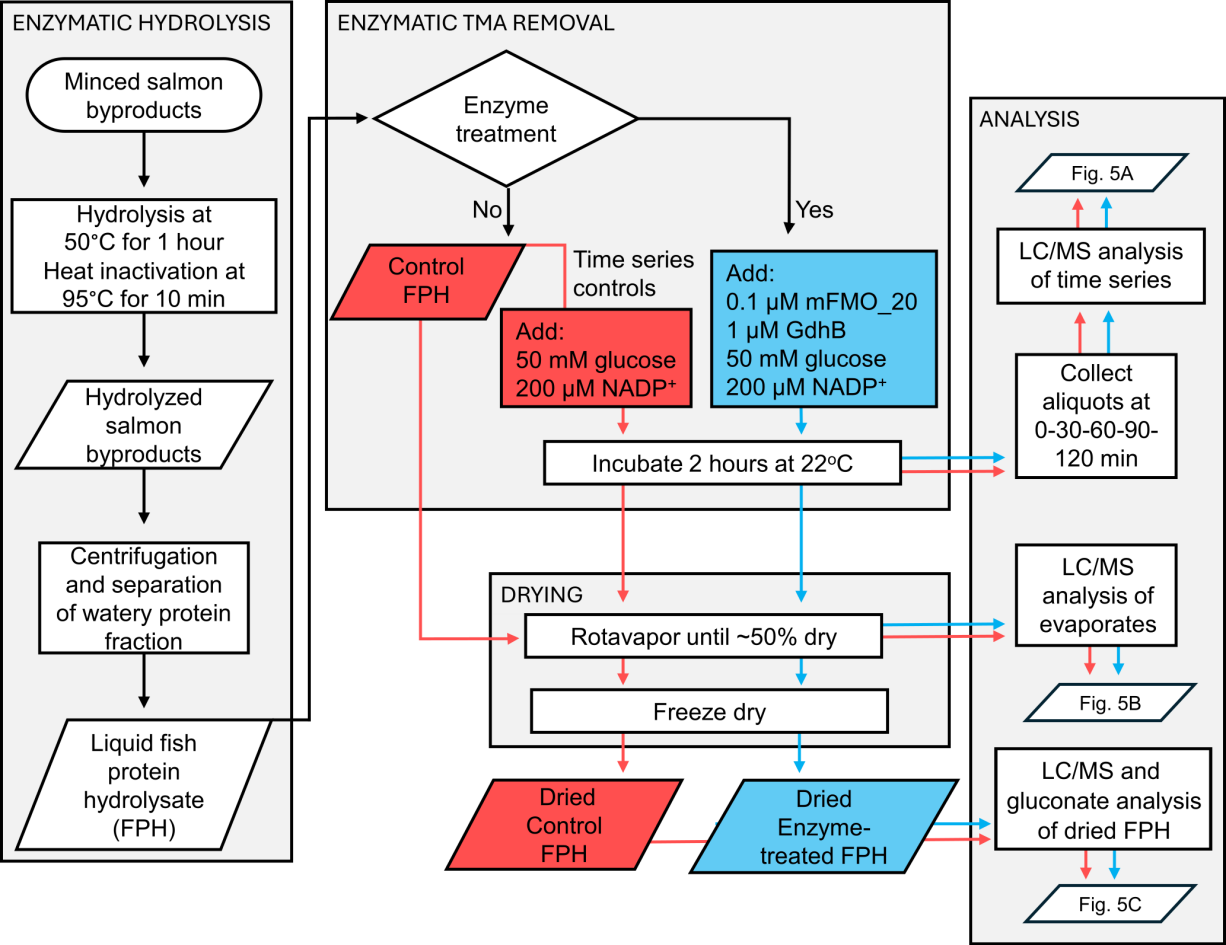
Production and evaluation of enzyme-treated FPH at lab scale. The rectangles indicate process steps and analyses, while diamonds show branching points, and the parallelograms contain intermediate and final products and indicate which figure contains the associated result. The flow of control FPH fractions is shown in red and enzyme-treated FPH fractions in blue. FPH, fish protein hydrolysate (salmon); LC/MS, liquid chromatography/mass spectrometry.

To demonstrate that the mFMO_20/GdhB enzyme cascade can deplete TMA in an industrially relevant protein hydrolysate, a lab-scale protease-driven hydrolysis of salmon heads and frames (byproducts) was performed (Fig. 4).

**Fig 5:**
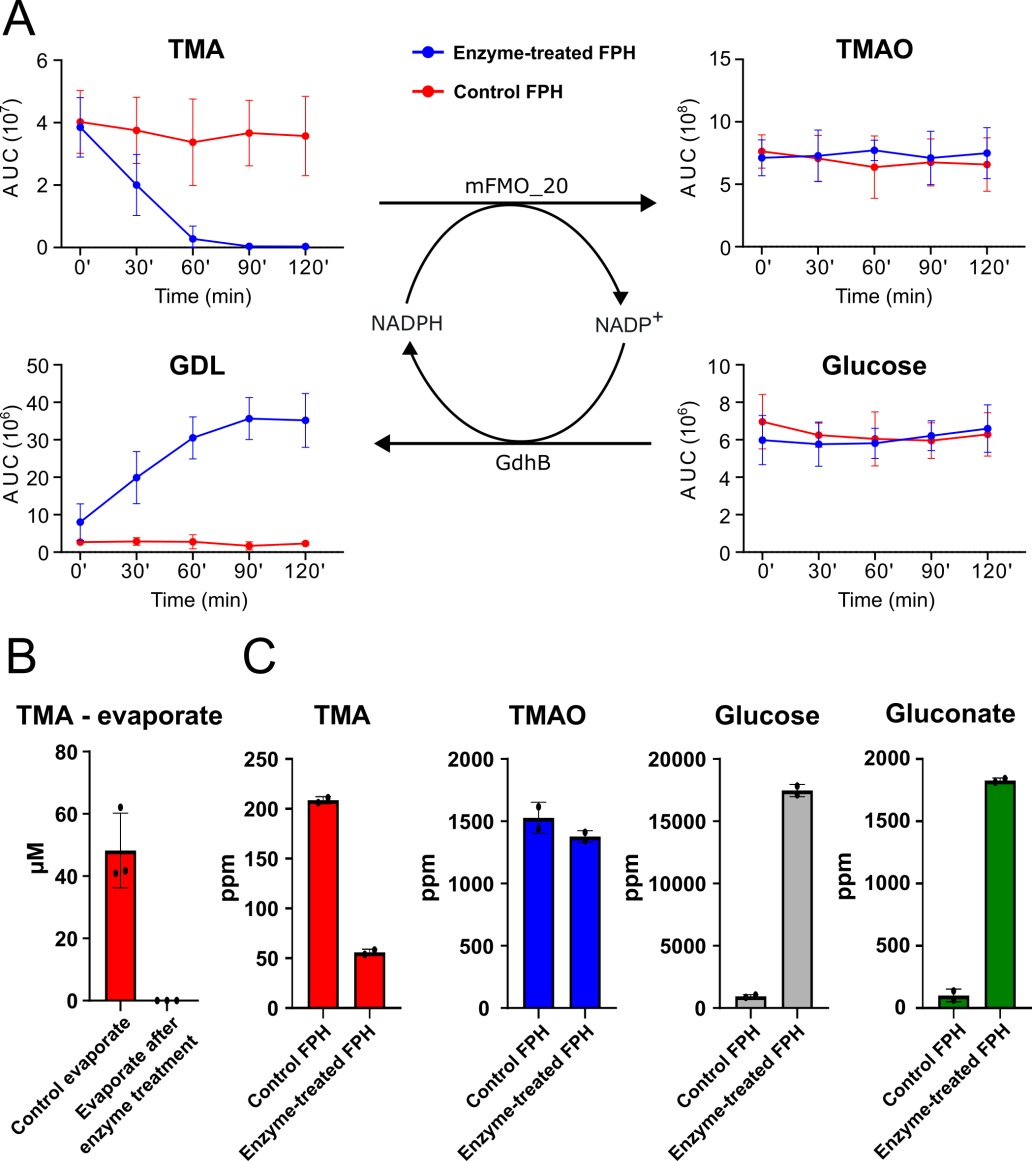
Cofactor recycling-driven TMA oxidation in salmon protein hydrolysate. (**A**) Time course analysis of the enzymatic TMA removal process. TMA, TMAO, glucose, and GDL were quantified at each time point by LC/MS. Blue: enzyme-treated FPH (presence of mFMO_20/GdhB); red: control FPH (absence of mFMO/GdhB). *n* = 6 for each time point (0, 30, 60, 90, 120 min) for control FPH and *n* = 4 for enzyme-treated FPH. Error bars show the standard deviation between replicate enzyme treatment batches. AUC, area under the curve. (**B**) TMA in the enzyme-treated and control FPH evaporates measured by LC/MS (*n* = 3). Error bars show the standard deviation between evaporate batches. (**C**) TMA, TMAO, glucose, and GDL were quantified by LC/MS in dried enzyme-treated FPH and control FPH; *n* = 2 injections of the same sample. Gluconate was quantified using an enzymatic approach and colorimetric detection against a standard curve (*n* = 2, measurements of the same sample). Error bars show standard deviation.

Whole, fresh salmon were filleted, and the heads and frames were minced in a meat grinder to produce the byproduct feedstock. This mince was mixed with 50% water (wt/wt) and 0.5% Alcalase 2.4L (vol/wt biomass), a commercial subtilisin endoprotease with broad specificity. After hydrolysis and heat inactivation of the protease, the sample was centrifuged to isolate the water-soluble fraction containing hydrolyzed peptides, hereafter referred to as FPH. To protect the added mFMO and GdhB enzymes from proteolytic cleavage and lipid interference, they were introduced only after hydrolysis and centrifugation, along with glucose and NADP^+^ (Fig. 4). We obtained a total of 4,860 mL liquid hydrolysate, which served as the substrate for enzymatic TMA removal treatment (Fig. 4, top middle box). The dry matter content was estimated to be 9.3%. LC/MS analysis of FPH samples collected during the enzymatic TMA removal process showed that treatment with mFMO_20 and GdhB depleted TMA in a time-dependent manner, reducing it to less than 1% of the initial intensity (Fig. 5A; Table S5). The TMA intensity was unchanged in controls where enzymeswere absent. The TMAO intensity was stable throughout the time course, likely reflecting the high intrinsic TMAO content in the freshly prepared FPH. The glucose concentration also remained stable, as it was added in great excess. GDL intensity increased over time in parallel with the reduction of TMA, confirming successful cofactor regeneration. The final dried control and enzyme-treated FPHs were prepared by evaporation until partially dried, followed by freeze-drying until completely dry. Analysis of the evaporate (Fig. 5B; Table S6) revealed approximately 50 µM TMA remaining in the control FPH, while no TMA was detected in the enzyme-treated evaporate. This demonstrates that although drying may assist in TMA removal, it is not sufficient to fully deplete it. Quantification of TMA in the dried FPHs showed that 58 ppm remained in the mFMO_20/GdhB-treated FPH, whereas 208 ppm was retained in the control (Fig. 5C). Hence, the enzyme treatment oxidized approximately 75% of the TMA content remaining after evaporation and drying. A previous study reported that the application of nano- and diafiltration reduced TMA from 700 to 100 ppm in cod FPH and from 400 to 100 ppm in salmon FPH (15). Reaching a level of 100 ppm was associated with improved TMA taste intensity.

We did not observe an increase in TMAO concomitant with the decrease in TMA in the enzyme-treated FPH. The 150 ppm TMAO produced may not make enough of a difference to be detected between samples, given that the TMAO concentration was measured between 1,344 and 1,615 ppm, and the variation between replicate injections was between 4.9% and 12.2% (Table S6). We did not detect GDL in the enzyme-treated FPH, consistent with the rapid rate of spontaneous GDL hydrolysis (43). Instead, gluconate was quantified using the same enzymatic colorimetric assay applied in the model reactions (Fig. 3), confirming that glucose oxidation occurred, and that cofactor recycling was maintained throughout the process (Fig. 5C). Together, these results demonstrate that the mFMO_20/GdhB enzyme cascade effectively reduces TMA levels in salmon protein hydrolysates under conditions relevant to industrial processing.

### Reduction of TMA levels in FPH assessed by sensory panels

**Fig 6:**
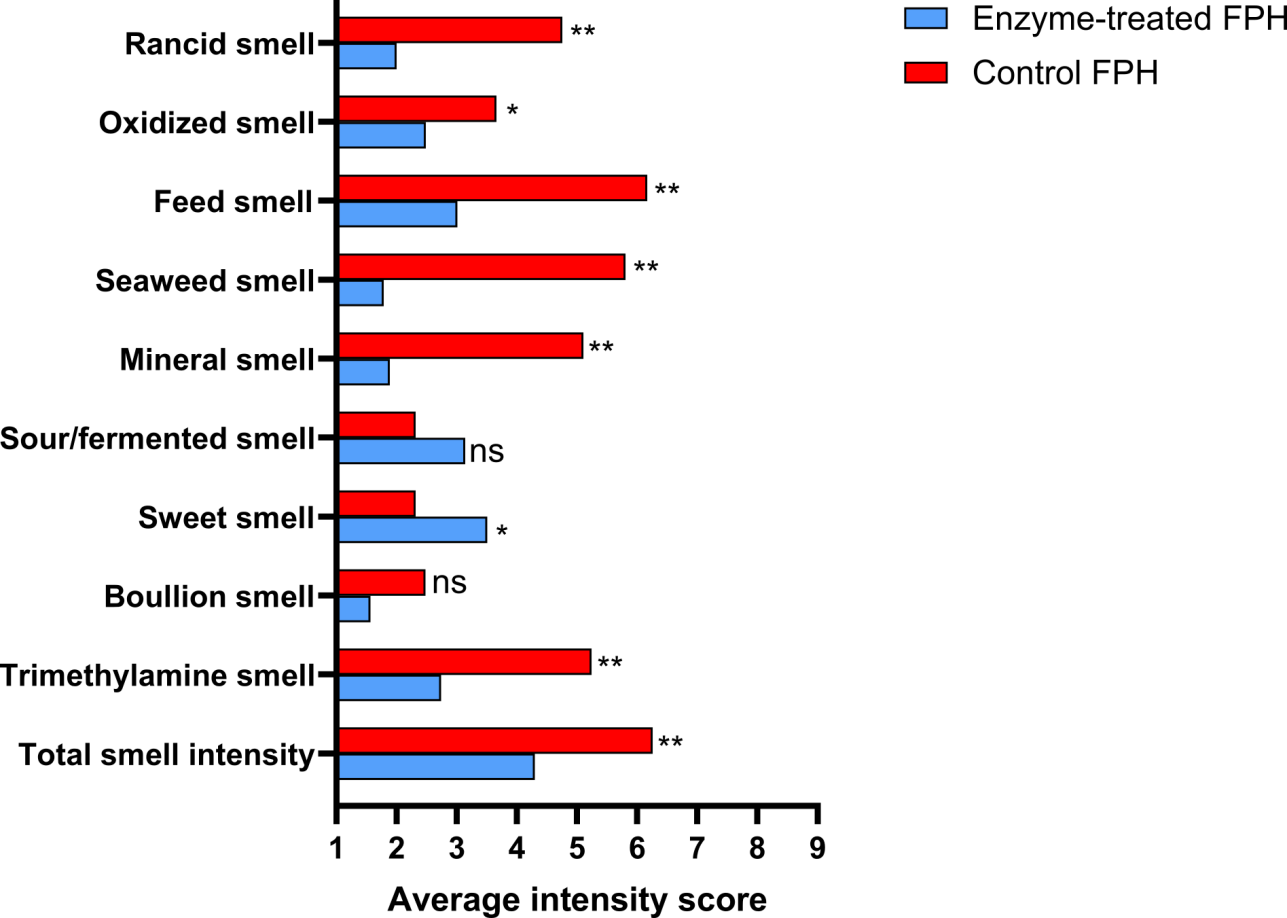
Assessment of fish protein hydrolysates by a trained sensory panel. Each sensory panel member (*n* = 8) scored the intensity of each dried hydrolysate twice on 10 odor characteristics in a blinded and randomized order. Enzyme-treated FPH and control FPH indicate the presence and absence of mFMO_20/GdhB treatment, respectively. Statistical significance was tested using ANOVA and Tukey’s test for multiple comparisons. **: *P* < 0.01, *: *P* < 0.05, ns: *P* > 0.05. See Table S3 for a description of each attribute.

To assess whether the reduction in TMA measured by LC-MS corresponded to a decrease in odor intensity, a trained sensory panel evaluated multiple odor attributes of the dried control and enzyme-treated FPHs. The evaluation included TMA and overall smell intensity, rated on a 9-point scale (Fig. 6; Table S7). The assessors detected significantly lower intensities in nine of the ten attributes for the enzyme-treated FPH. The TMA odor was perceived to be significantly lower by 2.5 units, which is in line with the observed 75% reduction in TMA levels (Fig. 5). The sensory panel is trained to detect TMA as distinct from other aromas, such as rancid or seaweed odors. Hence, the reduction in other sensory attributes is probably not caused by the reduction in TMA levels and may instead reflect enzyme activity on other compounds or effects from glucose. Notably, the enzyme-treated FPH scored significantly higher on sweet odor, likely due to the added glucose absent in the control. Both native mFMO, which served as the basis for mFMO_20 used in this study, and FMO from *Methylocella silvestris* have been reported to oxidize compounds beyond TMA, including dimethylamine, methimazole, indole, and dimethyl sulfide (19, 21). Although off-target oxidations by mFMO_20 have not yet been investigated, the oxidation of unknown metabolites may have influenced the olfactory profile of the FPH.

**Fig 7:**
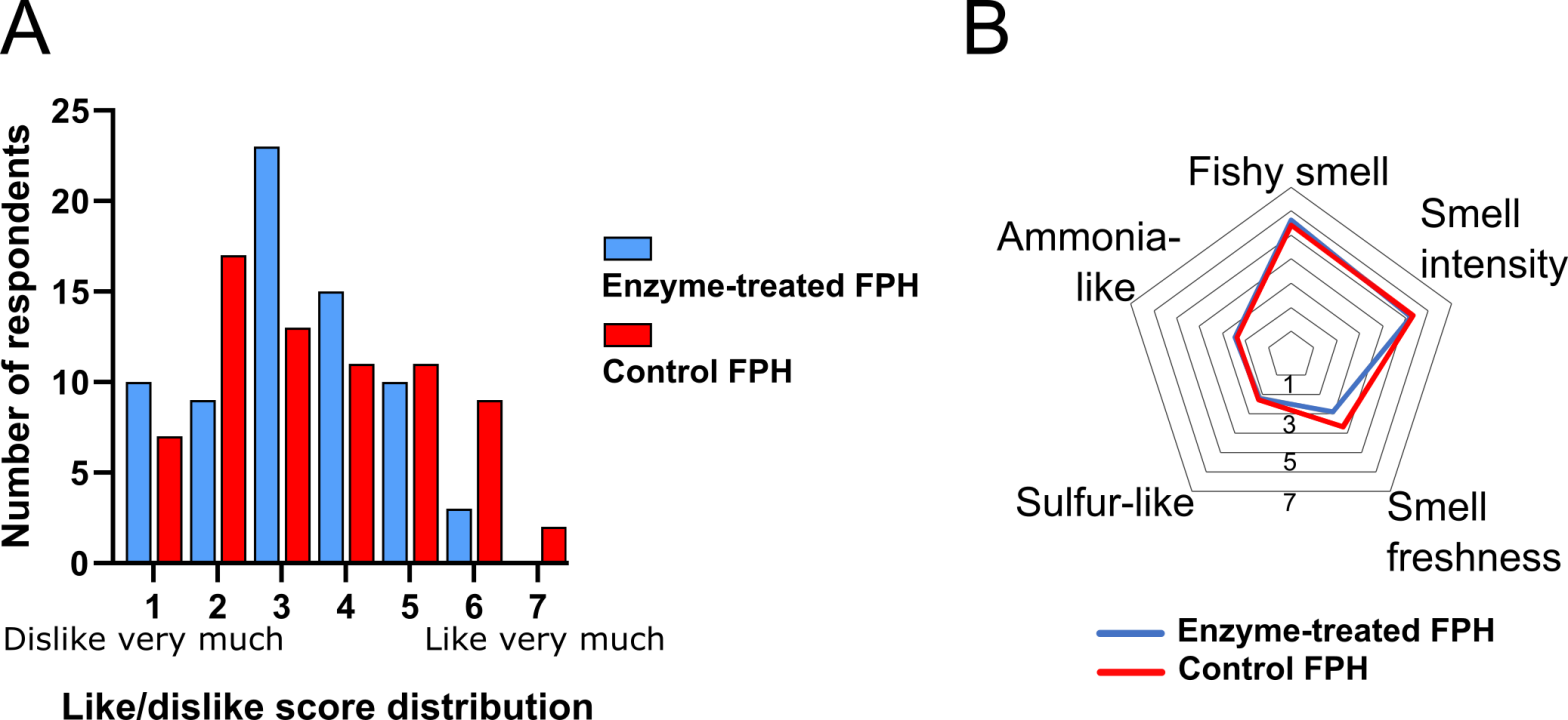
Sensory assessment of enzyme-treated FPH by a consumer panel. (**A**) Consumer acceptability test with distribution of smell perception scores. Participants (*n* = 70 participants) rated the smell of each hydrolysate on a 7-point scale, where 1 corresponds to “dislike very much” and 7 to “like very much.” The description of the scale is given in Table S8. (**B**) Average intensity scores were obtained from consumer panelists (*n* = 70) who rated the smell of each hydrolysate based on five attributes: fishy smell, ammonia-like smell, sulfur-like smell, smell freshness, and smell intensity. Ratings were given on a 7-point scale, where 7 indicated the highest intensity. Descriptions of the attributes are given in Table S9.

The ultimate aim of the TMA remediation process is to reduce TMA levels in FPH sufficiently to achieve consumer acceptance. To evaluate whether this reduction translates into improved consumer acceptance, 70 participants aged 24–59, of whom 61% were women, were recruited from the Province of Barcelona, Spain, for preference and acceptance testing of the enzyme-treated, dried FPH (Fig. 7). Participants evaluated acceptance, using a 7-point hedonic scale ranging from “dislike very much” (1) to “like very much,” and rated smell intensity on a 7-point scale from lowest (1) to highest (7).

The intensity attributes assessed were fishy smell, ammonia-like smell, sulfur-like smell, freshness, and overall smell intensity. Both the control and enzyme-treated FPHs showed similar acceptability and attribute intensities. The enzyme-treated FPH had an average acceptability score of 3.2, while the control scored 3.5, with a median of 3 for both (Fig. 7A). Average scores for fish smell attributes were not markedly different between the samples (Fig. 7B). Respondents were also asked to rank the samples by preference. Of the 69 respondents who indicated a preference (one did not), 42 (60.9%) preferred the control FPH, while 27 (39.1%) favored the enzyme-treated FPH. We conclude that the consumer panel did not perceive a difference between the enzyme-treated and control FPHs. The discrepancy between the two panels suggests that trained assessors are more sensitive to subtle changes in specific odor attributes in FPH due to their calibration and experience. The absolute magnitude of change observed by trained assessors may still be below the perceptual or hedonic relevance threshold for consumers. Consumers often require a larger perceptual difference, known as the just-noticeable difference, before they detect or translate such differences into liking (50, 51). This aligns with literature showing that untrained consumers frequently fail to detect or articulate changes in specific odorants unless these differences exceed the perceptual relevance threshold, particularly in complex food matrices (51–53). In our study, although the trained panel detected a significant decrease in TMA-related odor intensity, the overall odor profile of fish hydrolysates is driven by a complex mixture of volatile compounds. Therefore, the reduction in TMA may have been insufficient to shift the overall perceptual impression or hedonic evaluation by the consumer panel. This highlights the importance of using both types of panels to gain a comprehensive understanding of sensory changes.

TMA content of seafood and its raw materials varies with fish species and freshness. TMA is reported to vary between 0.3 g/kg wet weight (54) and 0.7 g/kg dry weight (15) in cod FPH, and between 0.4 g/kg (15) and 1.1 g/kg dry weight (17) in salmon FPH. Converting 1 g of TMA in FPH would require over 12 g of the reduced cofactor, NADPH, and would thus be economically prohibitive.

The final TMA concentration of the mFMO_20/GdhB-treated FPH (58 ppm) remains five orders of magnitude higher than the reported human detection threshold for TMA (0.00021 ppm vol/vol) (55). This suggests that the TMA smell would be detectable even after enzyme treatment. While removing TMA was expected to improve organoleptic properties by reducing TMA-related sensory attributes, the residual TMA level indicates that further optimization is needed. Additional experiments are needed to investigate if cofactor recycling can sustain the mFMO catalysis for longer and fully deplete TMA. A GdhB-driven NADPH regeneration system sustained the production of (R)-4-chloro-3-hydroxybutanoate ethyl for 5 h (34). However, a continuous reaction may depend on factors, such as NADP^+^ dimerization or isomerization, causing cofactor loss (25) or product inhibition.

Although TMA is widely recognized as a key contributor to the characteristic odor of fish and FPH (8), it is likely not the sole determinant of what is perceived as fishy smell. The overall sensory perception is likely influenced by a complex mixture of volatile organic compounds, including aldehydes, ketones, other amines, and sulfur-containing molecules, and concentrations and volatility also play a role (56, 57). A combination of targeted and untargeted approaches, for example, filtration (15) and evaporation (Fig. 5B) combined with enzyme treatment, may be needed to obtain FPH products that are acceptable to end users. Extended reaction times or increased enzyme dosage could be explored to further reduce TMA levels and weighed against the economic implications. It remains uncertain whether reducing TMA concentrations to imperceptible levels alone will sufficiently diminish the overall fishy smell.

### Conclusion and future opportunities

We have shown that an enzyme cascade combining mFMO and GdhB serves as a targeted approach to reduce TMA content in FPH, while lowering costs through efficient cofactor recycling. Using a combination of quantitative instrumental and sensory approaches, our results demonstrate both a decline in TMA levels and a corresponding reduction in TMA-derived sensory attributes. These results suggest that, with further optimization of reaction time and conditions, enzymatic treatment has the potential to achieve greater depletion of TMA than untargeted approaches. All of the experiments described herein were performed with salmon hydrolysates, and the mFMO/GdhB cascade remains to be tested with other TMA-containing protein hydrolysates. Exploring the feedstock scope of TMA-metabolizing enzymes to more classes of byproducts is thus an important follow-up step. To develop palatable FPH products, further research is needed to unravel the role and complexity of odor-contributing components, as the mechanisms underlying fishy smell formation are not yet fully understood. Exploring approaches to produce FPH that meet end-user acceptance offers an avenue for future investigation.

## MATERIALS AND METHODS

### Fermentation and purification of enzymes

mFMO_20 and GdhB enzymes were expressed in *Escherichia coli* from a vector encoding a C-terminal His-tag. GdhB additionally carries an N-terminal SpyTag (58). GdhB was expressed in 10 L 2YT medium (16 g/L tryptone, 10 g/L yeast extract, and 5 g/L NaCl) mFMO_20 was expressed in an 8 L medium with the following composition (in g/L unless otherwise specified): Na_2_HPO_4_·7H_2_O, 12.8; KH_2_PO_4_, 3.0; NaCl, 0.5; NH_4_Cl, 1.0; MgSO_4_·7H_2_O, 0.5; CaCl_2_·2H_2_O, 0.015; glucose, 0.5; ampicillin, 0.1; casamino acids, 10; glycerol, 4.25; L-leucine, 0.00005; trace metal solution, 1 mL. Composition of trace metal solutions (in g/L) FeCl_2_·4H_2_O, 1.5; ZnCl_2_, 0.07; CoCl_2_·6H_2_O, 0.19; MnCl_2_·4H_2_O, 0.1; CuCl_2_·2H_2_O, 0.02; NiCl_2_·6H_2_O, 0.024; Na_2_MoO_4_·2H_2_O, 0.036; H_3_BO_3_, 0.006; 25% HCl, 10 mL. Both enzymes were expressed in a 15 L turbine-stirred bioreactor (Chemap, Switzerland). Cultivation of both enzymes was started at 37°C ± 0.1°C. A pH meter (Mettler Toledo InPro 3030/120) was used to maintain the pH at 7.0 ± 0.1 with 1 M NaOH and 1 M H_2_SO_4_. The fermenter was operated at an overpressure of 0.2 bar and with a constant supply of sterile air (2.5 L/min) through the bottom sparger. Dissolved oxygen was maintained at a minimum of 30% of air saturation by regulating the stirrer speed during cultivation. Foaming was controlled by dropwise addition of an anti-foaming agent (A240, Sigma). Percentages of O_2_ and CO_2_ in the off-gas were measured continuously using a mass spectrometer (Prima Pro Process Mass Spectrometer, ThermoFisher), and the experimental data were acquired from LabVIEW 6 (National Instruments, USA). The batch phase started by inoculating the media with 500 mL of inoculum. When the optical density at 600 nm reached between 0.5 and 0.8, the cultivation temperature was reduced to 18°C–20°C, and the cultures were induced with 20% L-arabinose (final concentration in the broth 1 g/L) to initiate the enzyme production. When the fermenter’s off-gas CO_2_ concentration dropped, the batch phase was over. After completion of the batch experiment, the fermentation broth was harvested. The cell biomass was separated from the broth by centrifugation at 4,500 rpm for 20 min at 4°C. Cell paste was stored at −20°C. mFMO_20 was purified essentially as described (17) in 50 mM tris-HCl, pH 8.0, and 500 mM NaCl, while the buffer system for GdhB was 20 mM Na_2_HPO_4_–NaOH, pH 6.5, and 500 mM NaCl. Equilibration and washing buffers were supplemented with 10 and 50 mM imidazole, respectively. The elution buffer for mFMO_20 and GdhB contained 500 and 300 mM imidazole, respectively. The desalting buffers consisted of 500 mM NaCl and 10% glycerol. Cells were lysed in lysis buffer (equilibration buffer supplemented with 25 µg/mL lysozyme and 1 mM PMSF) by three freeze/thaw cycles, followed by sonication on ice (1/8″ probe, 70% amplitude, 10 s on and 20 s off, for a total of 2 min sonication time).

mFMO_20 was purified using an Äkta Pure chromatography system (GE Healthcare) with a 5 mL His-Trap column (GE Healthcare). The lysate was cleared for 30 min at 17,000 × *g* and loaded on the column using the sample pump. After washing with 20 column volumes (CVs) of wash buffer, the His-tagged protein was eluted by a stepped gradient of 5 CVs per step, at 35%, 50%, 65%, and 100% B. The eluate was collected in 2 mL fractions. Fractions that appeared yellow, likely due to bound FAD (59), were combined, and the buffer was exchanged to desalting buffer by sequential centrifugation using a 30 MWCO filter. Enzymes were aliquoted after concentrating to 6.9 mg/mL and stored at −80°C.

GdhB was purified using benchtop columns with 1.5 mL Ni-NTA resin (Qiagen). Cells were lysed, and the lysate was cleared in the same manner as for mFMO_20. The lysate was loaded onto equilibrated benchtop columns by gravity flow. Columns were washed with 20 CVs wash buffer and eluted in 2.5 mL elution buffer. After elution, GdhB was desalted using PD-10 columns (Cytiva) equilibrated with desalting buffer and eluted with 3.5 mL desalting buffer. The concentration at this point was between 1 and 3 mg/mL. GdhB was then aliquoted and kept at −80°C until use. Purity was analyzed by visual inspection using sodium dodecyl sulfate polyacrylamide gel electrophoresis. Concentration was measured using the 660 nm protein assay kit (Pierce), quantified against a standard curve of BSA.

### Enzyme assays and enzyme kinetics

Enzyme activity of mFMO_20 and GdhB was measured by spectrophotometrically monitoring the oxidation of NADPH or NADH, using the change in absorbance at 340 nm, and assuming a molar extinction coefficient of 6,220 M^−1^ cm^−1^. Enzymes were diluted to 1–10 µM and used at a final concentration of 50–1,000 nM, mixed with cofactor [NAD(P)H or NAD(P)^+^], and 100 µM TMA or 50 mM glucose or GDL. Activity was calculated as described (17). Briefly, all components except substrate were mixed and diluted to 1 mL in a polystyrene cuvette, using 50 mM tris-HCl, pH 8.0, and 50 mM NaCl as the reaction buffer, and the reaction was started by adding the relevant substrate (TMA, glucose, or GDL). Absorbance at 340 nm was monitored in a Cary UV-VIS photospectrometer over 1 min for mFMO_20 and 2 min for GdhB. The absorbance change per minute (positive for the NADP^+^ reduction reaction and negative for the NADPH oxidation reaction) was used to calculate the cofactor concentration change, assuming a molar extinction coefficient of NADPH of 6,200 M^−1^ cm^−1^. Activity is expressed as µM product formed min^−1^ µM enzyme^−1^, or min^−1^. Kinetic characterization of GdhB with constant NADP^+^ (100 µM) was performed with 0.1–1,000 mM glucose, in duplicate measurements. The substrate concentration (mM) versus velocity (min^−1^) was plotted, and the Michaelis-Menten parameters of *K*_m_ and *V*_max_ were calculated in GraphPad Prism. GdhB heat stability was assessed by first heating GdhB to 22°C (room temperature), 35°C, 40°C, 50°C, 57°C, 68°C, 66°C, or 70°C for 30 min, then measuring enzyme activity as described above.

### Cofactor regeneration enzyme assays

mFMO_20 (final concentration: 50 nM), GdhB (500 nM), cofactor (NADP^+^ or NADPH, 100 µM), glucose (50 mM), and TMA (500 µM) were diluted in 50 mM NaCl and 50 mM tris-HCl, pH 8.0. As reaction controls, either GdhB, NADP^+^, or glucose was omitted. The total reaction volume was 50 µL in a 1.5 mL microcentrifuge tube, and each condition was repeated three times. The reaction was started by the addition of substrate—glucose, or in the case of the condition with NADPH, by the addition of TMA. Reactions were briefly vortexed and centrifuged, and reactions were incubated at 25°C with light agitation for 1 h. The reactions were stopped by the addition of 450 µL 100% LC/MS-grade acetonitrile, vortexed, and stored at −20°C until analysis by LC/MS.

### FPH production by enzymatic hydrolysis

Two fresh, gutted salmon (10.186 kg) were purchased (Meny, Bergen Storsenter) and filleted. The remaining byproducts consisted of 2.430 kg of salmon heads, backbones, fins, tails, and skin (giving a byproduct fraction of 23.9%). Byproducts were minced and frozen at −20°C. Enzyme-assisted hydrolysis was performed in a customized bioreactor (H.E.L. automate, UK) by mixing 250 g thawed, minced byproduct with 250 mL water per reaction vessel, and heating to 50°C before adding 0.5% (wt/wt) Alcalase 2.4L (Sigma-Aldrich) and allowing hydrolysis to proceed for 1 h at 50°C. The enzymes were heat-inactivated at 90°C for 10 min. Insoluble material was removed by centrifugation at 5,000 × *g* and 15,000 × *g* for 20 min at 4°C. The water phase was separated from the oil phase using a separation funnel. The water phase was filtered through a 7 µm filter paper, then through 1.2 and 0.45 µm cellulose acetate filters. The filtered water phase was stored at −20°C.

### Enzyme treatment of FPH

For enzyme treatment, filtered FPH (in total 2.4 L, from 8 × 300 mL batches) was mixed with mFMO (final concentration: 100 nM), GdhB (final concentration: 1 µM), glucose (final concentration: 50 mM), and NADP^+^ (final concentration: 200 µM), and allowed to react for 2 h at 25°C under agitation. Samples for LC/MS analysis (50 µL) were taken after 0, 30, 60, 90, and 120 min. In parallel with each batch, 30 mL of hydrolysate controls without enzymes, cofactor, or glucose were incubated for time point controls. To dehydrate the hydrolysate, it was dried in a Rotavapor set to 60°C and 150–125 mbar of vacuum, until the volume was 14%–32% of the starting volume. The hydrolysate was further dried to completion in a freezer dryer, and the batches were combined and ground to a powder using a mortar and pestle. The non-enzyme-treated hydrolysate (2.05 L), dried in seven batches of approximately 300 mL, was dehydrated in the same manner.

### Analytical evaluation of enzyme assays and FPH by LC/MS

TMA conversion and the glucose dehydrogenase reaction were monitored by LC/MS. To prepare samples for LC/MS analysis, a 50 µL sample was mixed with 450 µL 100% acetonitrile and vortexed, then centrifuged for 20 min at 17,000 × *g* and 4°C. A volume of 75 µL of the supernatant was then transferred to an autosampler vial and kept at 4°C until analysis. The standards were prepared by serial dilution in 90% acetonitrile. The dried FPH was diluted to 1 mg/mL in 90% acetonitrile before clearing.

The mobile phase was 10 mM ammonium acetate, pH 8.0. Buffer A had 3% acetonitrile, and buffer B had 90% acetonitrile. The column was an ACQUITY Premier BEH Amide VanGuard FIT HILIC column (Waters), with particle size 1.7 µm and internal dimensions of 2.1 × 100 mm, fitted with a 2.1 × 5 mm guard column. A 3 µL sample or standard was loaded. The method took 14 min and used a 0.2 mL/min flow rate, starting at 85% B, going to 80% over 1.6 min, then to 40% at 10 min, and holding at 40% until 12.7 min, while final conditioning at 80% B happened between 12.8 and 14 min. An Orbitrap Q-Exactive (Thermo Fisher) was connected online to a Dionex UltiMate 3000 UHPLC system (Thermo Fisher). The mass spectrometer was operated in the positive mode for full ion scan, with scheduled switching to negative mode when GDL eluted at approximately 2.2 min. Quantification of the selected ions was performed in Excalibur Quant Browser (Thermo Fisher) by taking the area under the curves of the extracted ion chromatograms from samples and standards in defined RT windows (Table S2). The enzyme treatment of the hydrolysates was performed in batches, and these batches served as replicates for the statistical treatment of the quantified ions.

### Measurement of gluconate in enzyme assays and FPH

Enzyme assays (500 µL, *n* = 3 replicates) were prepared as described in “Cofactor regeneration enzyme assays,” above. After 1 h, reactions were stopped by heating to 80°C for 10 min to denature enzymes, followed by centrifugation for 5 min at 17,000 × *g* to remove proteins. Both GdhB (Fig. S2) and mFMO_20 (17) are heat-labile. The supernatant was transferred to a new tube, and 5 µL mFMO_20 (0.559 mM) and 100 µM TMA were incubated for 20 min at room temperature to scavenge any unreacted NADPH. This was followed by a second round of heating and centrifugation for protein removal. The supernatant, diluted 1:5 to stay within the standard curve range, was analyzed for gluconate using the D-Gluconate Assay Kit (Colorimetric) (Abcam, Cambridge, UK) (ab204703) according to the manufacturer’s instructions. Briefly, samples and gluconate standard were diluted in the sample matrix (tris-HCl, pH 8.0, 100 mM NaCl, 500 µM TMA, 50 mM glucose, 100 µM NADP^+^). A master mix was prepared using the kit components. A background control master mix was prepared using the same components while omitting the gluconate converter mix. A total of 50 µL samples were mixed with 50 µL master mix in a flat-bottom 96-well plate and incubated at 37°C for 40 min. Absorbance at 450 nm was read using a BioTek Epoch plate reader (Agilent). Background control absorbance was subtracted from sample absorbance, and gluconate was quantified against a standard curve from 0 to 227 µM gluconate. FPH-containing samples were dissolved in the matrix to 1% (wt/vol), and gluconate was quantified in the same manner as above.

### Sensory assessment by trained panel

Dried enzyme-treated and control FPH were assessed by a trained sensory panel at Nofima AS, using a quantitative descriptive analysis (ISO 13299:2016) and according to a generic descriptive analysis (51). The sensory panel consists of eight assessors, trained and satisfying the requirements of ISO 8586-1:2012. The assessors are continually trained on a variety of products and samples with differing sensory characteristics and different sensory methods. The rooms where the test was conducted were built according to ISO 8589:2007, and have individual judging booths, standardized lighting, and its own ventilation system. In a pretest, the assessors evaluated the samples and the agreed attributes on a comparison combination design. For the main test, the assessors were presented with the dried hydrolysates (either control or enzyme-treated), blinded as to which sample was which. Samples were served in a blocked and randomized fashion to each assessor. One gram of the sample in white cups with metal lids was served. They were evaluated on the smell parameters of total smell intensity, trimethylamine smell, bouillon smell, sweet smell, sour/fermented smell, seaweed smell, feed smell, oxidized smell, and rancid smell. The parameters are described in Table S3. Each assessor scored the intensity of the samples 1–9 for each parameter, with 1 being the least intense and 9 being the most intense. Panel averages were compared by ANOVA, and correction for multiple testing was done by Tukey’s test. The difference between control and enzyme-treated FPH was deemed significant if the corrected *P* value was <0.05.

### Consumer recruitment for sensory testing

A total of 70 adult consumers were recruited from among the employees of Leitat Technological Center, located in the Province of Barcelona (Spain), to take part in a sensory evaluation focused on odor perception. Recruitment was carried out internally by sending an email invitation to staff members through the organization’s internal mailing system. The email included a brief description of the study’s objective, the nature of the sensory evaluation (limited to smelling two samples), and the criteria for participation. Eligible participants were adults (≥18 years) with no known olfactory impairments. Employees interested in participating were directed to an online registration form, which collected demographic information (age, gender, location), verified eligibility, and allowed individuals to indicate preferred time slots. Although no strict quotas were applied, efforts were made to ensure demographic diversity among the participants. Before the evaluation, all participants signed an informed consent form, in compliance with ethical research standards approved by the relevant institutional review board.

### Consumer panel evaluation

During the sensory session, each participant was presented with two blinded hydrolysate samples contained in odor-isolated vessels. Sample A (enzymatically treated) and sample B (no treatment). Both samples have been prepared under the same conditions, ensuring uniformity in quantity and presentation, and have been given to the panelists in a room at a constant temperature and isolated from external odors to avoid interferences. Panelists evaluated specific odor-related parameters: fish smell, smell intensity, smell freshness, sulfur-like smell, and ammonia-like smell, using intensity and acceptability scales of seven points. The parameters are described in Table S4, and the intensity descriptions are in Table S5. A preference test between samples was also performed (60). Consumers were asked to rank the samples in order of preference. To obtain the ranking of each sample, each rank position was multiplied by the number of consumers who had selected it, and the sum of the rankings of each sample was calculated. Low values in the rank sum of samples indicated that the sample has mainly been ranked in the first order of preference.

## SUPPLEMENTAL MATERIAL

Supplemental material aem.01242-25-s0001.docx Fig. S1 and S2; Tables S1, S2, and S7 to S9.

Tables S3 to S6 aem.01242-25-s0002.xlsx LC/MS quantification results presented in the figures.

ASM does not own the copyrights to Supplemental Material that may be linked to, or accessed through, an article. The authors have granted ASM a non-exclusive, world-wide license to publish the Supplemental Material files. Please contact the corresponding author directly for reuse.
