# Supplementary material for: Monooxygenase-dehydrogenase cascade for sustained enzymatic remediation of TMA in salmon protein hydrolysates
Source: Appl Environ Microbiol. 2026 Feb 3;92(3):e01242-25. doi: 10.1128/aem.01242-25 (PMC12997807; doi:10.1128/aem.01242-25)
Supplement: Supplemental material — Fig. S1 and S2; Tables S1, S2, and S7 to S9. [file aem.01242-25-s0001.docx]

**Supplementary figures and tables**


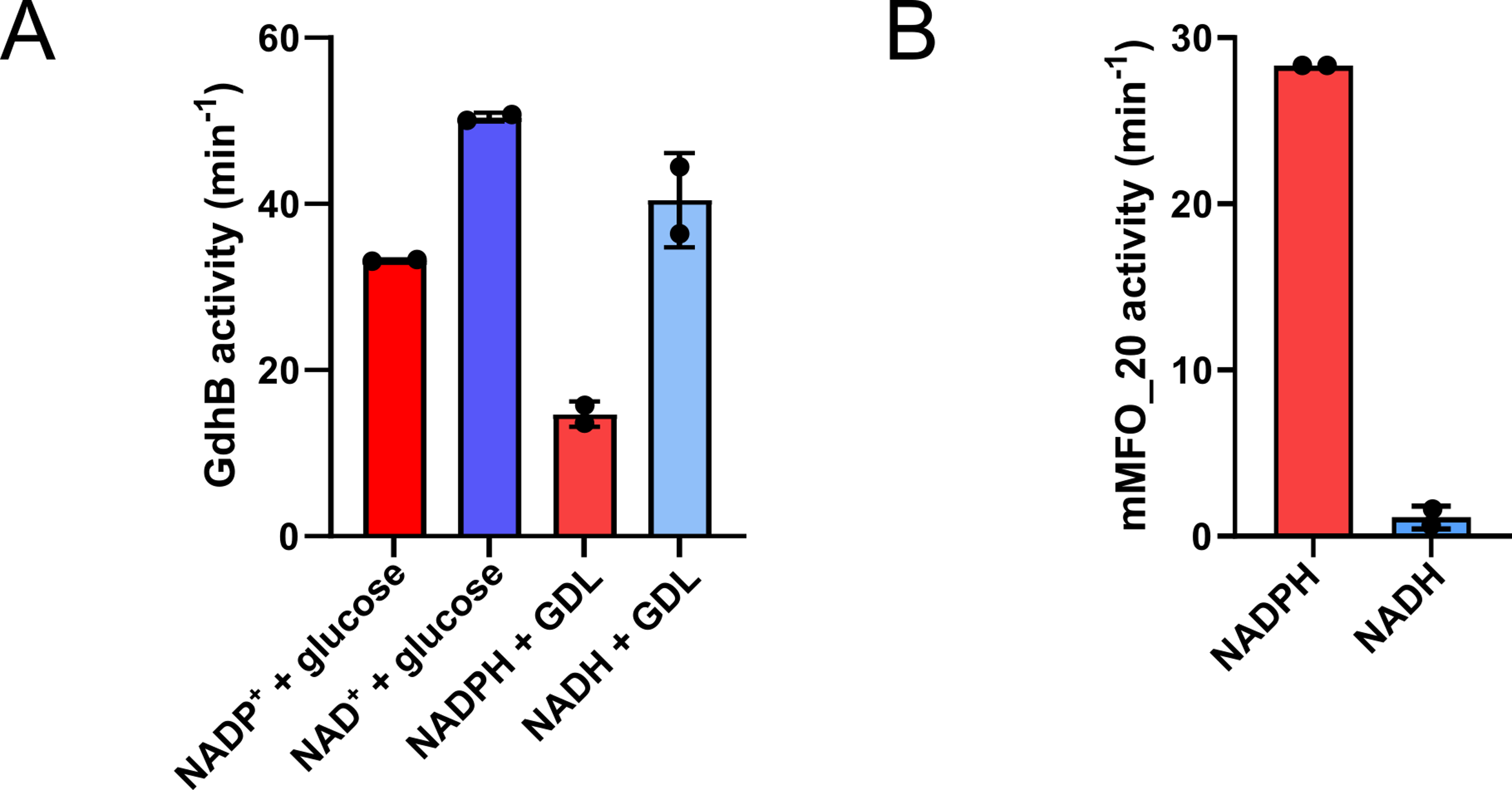


**Figure S1:** A) Activity of purified GdhB (500 nM) with glucose (50 mM) and the indicated oxidized cofactor (100 µM), and with the indicated reduced cofactor and GDL (50 mM), measured by absorbance change at 340 nm. B) Activity of purified mFMO_20 (50 nM) with 100 µM trimethylamine (TMA) and 100 µM reduced NADPH or nicotinamide adenine dinucleotide (NADH), measured by absorbance change at 340 nm.


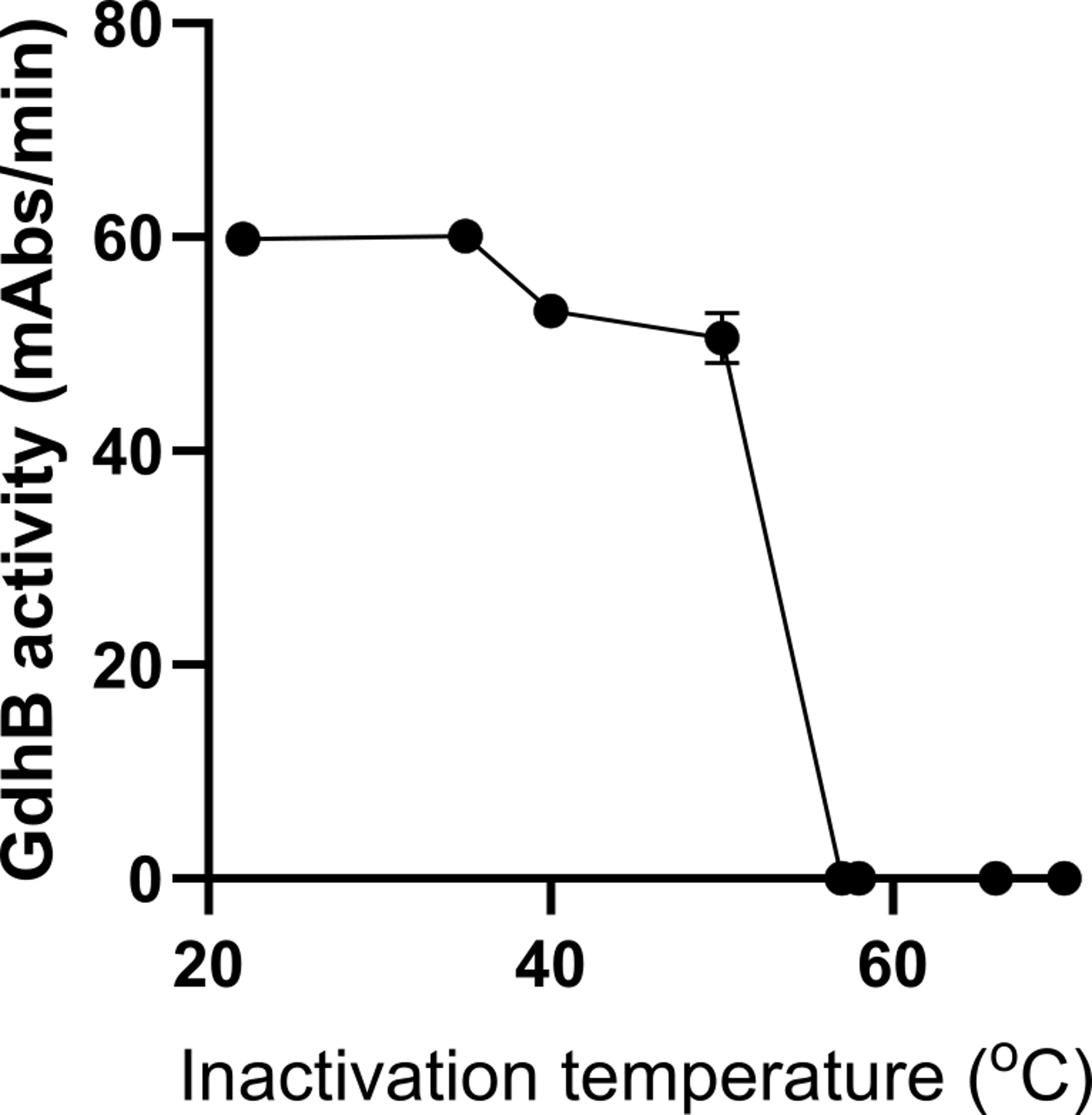


**Figure S2: Heat inactivation of GdhB.** GdhB (5 µM) was heated to the indicated temperature, then cooled on ice. Activity was then measured by absorbance change at 340 nm during an assay with 500 nM GdhB, 100 µM NADP^+^, 50 mM glucose, 100 mM NaCl, and 50 mM tris-HCl, pH 8.0.

**Table S1: Kinetic parameters of GdhB using glucose as a substrate. NADP^+^ concentration. Parameters of mFMO_20 are included for reference.**

| Enzyme | *V*_max_ (s^-1^) | *K*­­_m_ (mM) | Specificity constant (s^-1^ M^-1^) | Reference |
| --- | --- | --- | --- | --- |
| GdhB | 0.429 | 68.68 (glucose) | 6.25 | This study |
| mFMO_20 | 0.93 | 0.83 x 10^-3^ (TMA) | 1.11 x 10^6^ | Goris et al, 2023 |

**Table S2: Metabolites and related analytical information quantified by LC/MS.**

| Metabolite | Molecular formula | Mass (g/mol) | Ion  (m/z, charge) | Retention time  (minutes) |
| --- | --- | --- | --- | --- |
| TMA | C_3_H_9_N | 59.112 | 60.08160 (+H) | 3.82 |
| TMAO | C_3_H_9_NO | 75.11 | 76.07630 (+H) | 4.58 |
| Glucose | C_6_H_12_O_6_ | 180.156 | 198.09677 (+NH4) | 3.78 |
| GDL | C_6_H_10_O_6_ | 178.14 | 177.03991 (-H) | 2.17 |

**Table S3-S6: Quantification of selected ions by LC/MS.** Attached Excel sheet. Contains quantitation results and standard curves (Table S3: Standard curves for LC/MS quantification) of TMA, TMAO, glucose and GDL used in Figure 2 (Table S4: LC/MS quantification of analytes in *in vitro* enzyme assays) and Figure 4 (Table S5: LC/MS quantification of analytes in time curve and Table S6: LC/MS quantification of analytes in finished hydrolysates).

**Table S7: Description of smell criteria for dry hydrolysates, employed by trained sensory panel.**

| Parameter | Description |
| --- | --- |
| Total smell intensity | Intensity of all smells in the sample |
| Trimethylamine smell | The smell of trimethylamine (TMA) |
| Sweet smell | Related to a sweet smell |
| Sour/fermented smell | A fermented sour smell, spoiled (the smell of a sour dishrag) |
| Mineral smell | Related to smells of plaster, lime, chalk and dryness |
| Seaweed smell | Related to fresh and dried seaweed and greens, green tea |
| Feed smell | Related to the smell of fish feed |
| Oxidized smell | Related to an oxidized smell which reminds you of dust, drawers and cardboard |
| Rancid smell | The intensity of all rancid smells (grass, hay, candle wax, paint, tallow, soap) |

**Table S8: Hedonic scale description, used in the consumer panel.**

| Score | Acceptability description |
| --- | --- |
| 1 | Dislike very much |
| 2 | Dislike moderately |
| 3 | Dislike slightly |
| 4 | Neither like nor dislike |
| 5 | Like slightly |
| 6 | Like moderately |
| 7 | Like very much |

**Table S9: Description of odor parameters used in the consumer panel.**

| Odor parameter | Description |
| --- | --- |
| Fishy smell | Degree to which a fishy odor is perceived |
| Smell intensity | Overall intensity of the sample’s odor |
| Smell freshness | Perception of how fresh the smell is (pleasant, non-stale) |
| Sulfur-like smell | Perception of any sulfur-related notes (rotten egg, pungent) |
| Ammonia-like smell | Detection of ammonia or any sharp chemical odors |
